# Current strategies for mobilome research

**DOI:** 10.3389/fmicb.2014.00750

**Published:** 2015-01-22

**Authors:** Tue S. Jørgensen, Anne S. Kiil, Martin A. Hansen, Søren J. Sørensen, Lars H. Hansen

**Affiliations:** ^1^Section of Microbiology, Department of Biology, University of CopenhagenCopenhagen, Denmark; ^2^Environmental Microbiology and Biotechnology, Department of Environmental Science, Aarhus UniversityRoskilde, Denmark

**Keywords:** mobilome, plasmidome, PacBio, plasmid, horizontal gene transfer, metagenomics, mobile genetic elements, MGE

## Abstract

Mobile genetic elements (MGEs) are pivotal for bacterial evolution and adaptation, allowing shuﬄing of genes even between distantly related bacterial species. The study of these elements is biologically interesting as the mode of genetic propagation is kaleidoscopic and important, as MGEs are the main vehicles of the increasing bacterial antibiotic resistance that causes thousands of human deaths each year. The study of MGEs has previously focused on plasmids from individual isolates, but the revolution in sequencing technology has allowed the study of mobile genomic elements of entire communities using metagenomic approaches. The problem in using metagenomic sequencing for the study of MGEs is that plasmids and other mobile elements only comprise a small fraction of the total genetic content that are difficult to separate from chromosomal DNA based on sequence alone. The distinction between plasmid and chromosome is important as the mobility and regulation of genes largely depend on their genetic context. Several different approaches have been proposed that specifically enrich plasmid DNA from community samples. Here, we review recent approaches used to study entire plasmid pools from complex environments, and point out possible future developments for and pitfalls of these approaches. Further, we discuss the use of the PacBio long-read sequencing technology for MGE discovery.

## INTRODUCTION

Thanks to the enormous adaptability of pathogenic bacteria in clinical settings, public interest in microbiology has never been greater. Bacterial plasmids are the main basis of this adaptability and provide a platform for the recombination and transfer events that allow genes to spread horizontally between genera (Sørensen et al., 2005). This adaptability enables bacteria to acquire accessory genes such as antibiotic resistance genes and entire genetic pathways, e.g., encoding degradation of pesticides and other xenobiotic compounds in various environmental settings (Izmalkova et al., 2006; Dealtry et al., 2014).

Until recently, the study of plasmids in the environment was based on indicators of diversity using indirect and relative methods such as gradient gel resolution of PCR products, quantification of groups of incompatibility using qPCR, Southern blotting, or analysis of plasmids from cultivated transconjugants (Götz et al., 1996; Smalla et al., 2000). The recent revolution in sequencing technologies has provided researchers with a more nuanced view of mobile genetic elements (MGEs) in complex environments, since the cultivation and primer biases that previously limited research have been replaced by a different set of biases, yielding different insights. The sequencing based approaches aim to analyze entire MGE pools of communities or biological niches rather than select groups and are known by various names, such as mobilomics (Jørgensen et al., 2014), metamobilomics (Li et al., 2012; Norman et al., 2014), plasmidomics (Kav et al., 2012) plasmid metagenomics (Palenik et al., 2009; Sentchilo et al., 2013), and viromics in the study of viruses (Lopez-Bueno et al., 2009). Though their applications differ slightly, these are, in essence, based on similar strategies, achieve the same type of results, and are restricted by some of the same biases.

Here, we discuss the biases and possibilities of current approaches and attempt to predict the direction in which this research is moving, specifically in regards to emerging sample preparation techniques, sequencing technology, and bioinformatics. The above methods are referred to collectively as mobilomics in this perspective article as not only gene-carrying plasmids, but also other circular elements such as phages and circular transposons are the target of these types of DNA purifications and as the increased mobility of these genetic elements relative to chromosomal DNA are pivotal for research interest in them.

## MOBILOME SAMPLE METHODOLOGIES

In recent publications, there have been several approaches to obtain mobilome samples from complex environments, with the four most influential being A Cesium Chloride Ethidium Bromide (CsCl-EB) -gradient ultracentrifugation (Sentchilo et al., 2013), B bioinformatical derivation of plasmid related contigs/genes (Palenik et al., 2009; Ma et al., 2012), C transposon aided capture (TRACA; Jones and Marchesi, 2006), and D degradation of linear DNA with exonuclease followed by multiple displacement amplification (MDA; Kav et al., 2012, 2013; Li et al., 2012; Jørgensen et al., 2014; Norman et al., 2014). In **Table 1**, the routes of the above studies are outlined, highlighting the similarity of approaches.

**Table 1:** Routes for analyzing plasmid diversity in recent publications.

Study	Input material	Sample treatment	DNA purification	DNA treatment	DNA amplification	Sequencing platform	Confirmation of circularity
Jones and Marchesi (2006)	Human feces	Nycodenz	Alkaline lysis	Exonuclease Transposon aided capture (TRACA)	Transformation, culturing	Sanger, primer walking	PCR
Ma et al. (2012)	Seawater	Filtration, FACS	Phenol–chloroform	–	MDA	454	Inverse PCR
Kav et al. (2012)	Cow rumen	Homogenization, centrifugation, filtration	Alkaline lysis and lysozyme-SDS	Exonuclease	MDA	Illumina GAIIx	–
Li et al. (2012)	Wastewater	Sedimentation, centrifugation	Plasmid prep kit	Exonuclease	MDA	454	–
Sentchilo et al. (2013)	Sludge	Homogenization, centrifugation, pectinase	Alkaline lysis, acid phenol chloroform	CsCl-ethidium bromide density gradient ultracentrifugation	–	Sanger 454FLX	PCR
Jørgensen et al. (2014)	Rat cecum	RNAlater storage	Plasmid prep kit	Exonuclease	MDA	Illumina HiSeq2000 +454FLX	*In silico* and inverse PCR
Norman et al. (2014)	Wastewater	Filtration, centrifugation	Plasmid prep kit	Exonuclease, AGE size separation, electroelution	MDA	Illumina HiSeq2000	–

Approach A, CsCl-EB-gradient ultracentrifugation, is a classical and potent plasmid purification method, but the widespread use have diminished in the later decades after faster purification techniques became available in the early nineties. The method requires a large amount of starting material and specialized equipment, both of which are limiting factors for the use of this method (Sambrook et al., 1989). The advantage of this approach, though, is its potential to obtain large intact plasmids, a feat none of the other methods have been able to efficiently achieve. After careful DNA extraction and prolonged ultracentrifugation, supercoiled covalently closed circular DNA will stabilize and form a band in the centrifuge tube that can be harvested by needle puncture of the tube. This yields μg of plasmid DNA that can subsequently be sequenced. The accessory genes predicted on sequences resulting from CsCl-EB-gradient ultracentrifugation are largely unknown, pointing to the power of this method to capture a fraction of large plasmids that have not been studied before. In the only published study using this method (Sentchilo et al., 2013), a high fraction of 16S rDNA carrying chromosomal DNA was found in one of three samples, compared to the plasmid purities reported for the enzyme based method described below (approach D). Approach B, bioinformatical derivation of plasmid related genes/contigs, is regularly used and built into several bioinformatical pipelines such as MG-RAST (Meyer et al., 2008). This metagenomic-based approach is commonly used when mobility is not the focus of study as only a small fraction of metagenomic datasets stem from MGEs. Further, it relies heavily on database adequacy and is thus most useful for studying well-characterized environments. Approach C, TRACA, rely on inserting a transposon carrying a selective marker on MGEs that are then used to transform bacteria. The transformants can then be grown selectively in culture and the MGEs extracted. Method C was promising when published, but has not gained popularity, possibly owing to its low throughput and inability to capture large plasmids outside of the range of the method described below. Further, it relies on the ability of *Escherichia coli* to maintain the plasmid thereby presumably excluding most plasmids from identification. For the above reasons and due to the fact that no published studies in the last 3 years have utilized this system, the method will not be touched upon further in this perspective article.

**FIGURE 1:**
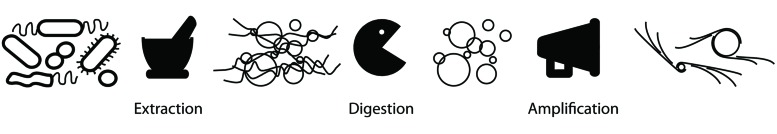
**Workflow for mobilome analysis as used in Kav et al. (2012), Li et al. (2012), Jørgensen et al. (2014), and Norman et al. (2014).** Initial careful DNA extraction is followed by digestion of linear and nicked DNA with exonuclease and unspecific but selective amplification of circular DNA using multiple displacement amplification (MDA).

Approach D uses exonuclease to degrade presumably nicked chromosomal DNA and subsequently the rolling circle type MDA in order to enhance the signal from circular DNA and produce sufficient quantities of DNA for library construction (workflow illustrated in **Figure 1**). This approach has enabled analysis of complete circular sequences and produced numerous very pure mobilome samples. The mobilome sample purity has been estimated by qPCR on the 16s rRNA gene, where purities of upward of 99% are not uncommon in published and unpublished data (Kav et al., 2013; Jørgensen et al., 2014). After sequencing, mapping of reads on the Arb-Silva database of ribosomal DNA has confirmed the accuracy of this method (http://www.arb-silva.de/, unpublished data). The estimation of chromosomal DNA contamination is particularly important as our extensive experience with building mobilome samples has led to acceptance of regular and seemingly stochastic failure in obtaining sufficiently low chromosomal contamination, often necessitating repetition of the entire procedure.

By analyzing DNA sequence data from mobilome samples, researchers have found a plethora of new, small plasmids and small plasmid fragments. Notably, though, very few plasmids larger than 10 kb have been completely assembled without using manual, PCR-based genome closing (Sentchilo et al., 2013; Jørgensen et al., 2014). Further, diversity of genes encoding plasmid stability (replication, mobilization, and toxin-antitoxin systems) outnumber diversity of accessory genes encoding, e.g., antibiotic resistance or substrate degradation, a striking difference from database plasmids that are often carrying multiple accessory genes (Kav et al., 2012; Jørgensen et al., 2014). Among the genes not related to essential plasmid functions, the proportion of genes encoding a protein with unknown function is high, often above 50% of predicted genes (Sentchilo et al., 2013). While the genetic composition of the found plasmids may, to some extent, reflect the reality in nature, it seems likely that known methodological biases contribute to this outcome. Thus, small plasmids are thought to be capable of carrying relatively few accessory genes and in every step of the predominant mobilome sample preparation (**Figure 1**), a new bias toward small plasmids is introduced (Jørgensen et al., 2014). One important such bias in mobilome sample construction is in the purification of DNA (**Table 1**, ‘DNA treatment’ and **Figure 1** ‘Extraction’ and ‘digestion’). Because larger plasmids will be subjected to greater shear forces than smaller ones when in solution, they are more likely to break, and thus become substrate in the following exonuclease treatment. Further, different methods of extraction are thought to be more effective depending on particular bacterial groups, for example the Nycodenz gradient centrifugation method where both over- and underrepresentation of phyla have been observed (Holmsgaard et al., 2011). Another example is the plasmid prep extraction used in, e.g., (Li et al., 2012; Jørgensen et al., 2014). This plasmid extraction method is optimized for *E. coli* and is thought to underrepresent bacteria with a more rigid cell wall structure. This extraction bias have been attempted to be countered by pooling DNA purified with different methods (Kav et al., 2012). MDA bias are described in detail below and presumed less important biases are described in more detail in a previous study from our group (Jørgensen et al., 2014).

The great advantage in using the enzyme based mobilome sample method depicted in **Figure 1** is the purity of the resulting plasmid DNA, a purity that allows researchers to categorize all resulting reads and contigs as plasmid derived. This situation contrasts that of metagenomes, where reads are assumed to stem from chromosomes unless distinct plasmid-like features such as plasmid replication protein can be found, implying a bias toward previously characterized sequences. The mobilome sample method, on the other hand, is free of such biases, which allows exploration of entirely novel classes of circular MGEs. Moreover, as the polymerase used for MDA is able to amplify circular sequences multiple times for each binding forming a linear, direct-repeat structure, complete closed plasmids from a single plasmid can, theoretically, be identified. The closing of sequences into circular units are important as they represent complete biological units with full genetic context as opposed to bits and pieces such as reads, contigs, and scaffolds which might lack important contextual information (Jørgensen et al., 2014).

## THE USE OF MULTIPLE DISPLACEMENT AMPLIFICATION IN PLASMID STUDIES

Multiple displacement amplification is widely used in single cell genomics and many other fields where the initial amount of DNA is insufficient for analysis. The method combines the tight binding and low error rate of the DNA polymerase from the phi29 phage with capped random primers causing strand displacement rather than digestion to produce up to 100 kb fragments with little sequence bias (Dean et al., 2001; Hutchison et al., 2005). The method has progressed from one-of-many amplification methods to a dominating position in unspecific amplification of DNA. The advantages are many: high yield, fast and easy preparation and the ability to re-amplify DNA with little to no sequence diversity loss, if the stock is running low (Dean et al., 2001; Hutchison et al., 2005). The amplification comes at a cost, however. In the case of plasmid amplification, the rolling circle mechanism allows the phi29 polymerase to selectively amplify circular molecules, but has long been hypothesized to skew abundance relation between individual circular molecules as each nucleotide on a short circular molecule is copied multiple times per polymerase binding, making quantitation efforts on MDA-based samples questionable at best. A recent publication have confirmed and quantified this important method bias (Norman et al., 2014). While efficient in suppressing chromosomal ‘background’ DNA in samples, the small circular element bias will also impair the discovery of important larger plasmids carrying interesting accessory genes (Hutchison et al., 2005; Norman et al., 2014).

## MOBILOME PREPARATIONS WITHOUT AMPLIFICATION

As MDA introduces a major bias in the mobilome sample preparation protocol (Norman et al., 2014), a natural consequence would be to omit the MDA step. Previously, this was not feasible because of input DNA quantity requirements to sequencing library construction, but with the emergence of enzyme-based DNA fragmentation, such as Nextera XT®, as little as 20pg DNA can be used as input, theoretically allowing the omission of MDA (Parkinson et al., 2012). The amount of DNA left after digestion and before MDA (**Figure 1**) is so minuscule that it is difficult to measure with standard methods such as Qubit HS fluorometry or spectrophotometry, and thus chromosomal contamination cannot be quantified prior to sequencing.

Although well documented, only one paper using the CsCl-EB-gradient method has documented success with library construction from unamplified mobilome samples to date. And unpublished results from our lab using the exonuclease-MDA method (**Figure 1**) suggest that the levels of chromosomal DNA in such samples are at least an order of magnitude higher than in MDA samples from the exact same DNA purifications, possibly owing to incomplete breakage/nicking of chromosomal DNA during purification. The high levels of chromosomal contamination in unamplified samples support the assumption that a major factor in achieving low chromosomal DNA content in mobilome samples is the MDA process itself. Contrary to expectations, sequencing of two unamplified mobilome samples has not yielded longer circular contigs, suggesting downstream pipeline shortcomings (unpublished data). These insufficiencies need to be investigated by more systematic testing and deeper sequencing. An alternative strategy to omitting MDA to include large plasmids in mobilome samples is size selection by gel electrophoresis and electro-elution (Norman et al., 2014). This approach aims to remove small plasmids before MDA, thus preventing them from claiming the lion’s share of rolling circle amplification. It is, however, yet to be explored fully with contemporary, sensitive, small volume methods, such as capillary electrophoresis, which minimizes cross-contamination and human error.

## REPEATED STRUCTURES PROBLEM AND LONG READ SEQUENCING TECHNOLOGY

A recurring problem in the assembly of complete circular plasmids from complex communities is the many recombination events leading to repeated structures (Conlan et al., 2014). Thus, if an interspersed repeat is longer than the read length, most assemblers such as velvet (Zerbino and Birney, 2008) or IDBA-UD (Peng et al., 2012) will not be able to resolve the repeat beyond scaffolding, even with the help of paired end information (Jørgensen et al., 2014). This prevents automated recognition of many full circle plasmids, as it breaks large circular structures into several contigs. An example of this is seen in the only publication to date that has succeeded in closing large plasmids from a complex environmental genetic pool (Sentchilo et al., 2013). Here, manual, PCR based genome closing was necessary to achieve the interesting results presented (Personal Communication). That PCR was needed for genome closing highlights both the ability of this method to successfully acquire information on large plasmids and the inability of assembly programs to automatically finish genomes.

Longer reads are seen by many as the solution to the repeat problems in plasmid sequencing. So far, only Pacific Biosciences (PacBio, Menlo Park, CA, USA) have made a commercial breakthrough with reads of up to 50 kb, though mostly much shorter in the range 1–10 kb. The technology has struggled with a poor quality of individual reads (low Phred score), but this could and should be corrected with either multiple readings of each fragment, high coverage or shorter, more reliable reads such as Illumina. Even with such correction, further problems with for PacBio plasmid sequencing are low output volume and the large amount of DNA required for sequencing (10–20 μg of DNA). Possibly, CsCl-EB-gradient ultracentrifugation could generate the required amounts of pure and unamplified plasmid DNA, but fruitful coupling of the two technologies remain to be shown.

A method to directly sequence plasmids on the PacBio platform with no library preparation and low quantity of input DNA has been suggested, but even in optimal conditions with a cultured mock community of two known plasmids has only been shown to yield 3.5% (479) plasmid derived reads from four PacBio RS runs (Coupland et al., 2012).

To test whether plasmid derived reads could be extracted from an MDA mobilome sample, we have submitted a well-defined sample (Jørgensen et al., 2014) to PacBio RS sequencing and analyzed the results by close manual dotplot inspection after error correction with Illumina reads and assembly with MIRA (Chevreux et al., 1999) (see supplementary materials and methods). The dot-plots seen in supplementary materials Figures S1A–C represent typical circular contigs, with A, representing a rare circular sequence of app. 2.5 kb and B, and C, representing the much more common multiple short repeats structure. From the assembly of one PacBio RS run yielding 24,711 reads with an N50 of 4392 nt, 12 entire plasmids smaller than eight kb could be identified whereas the rest of the potential circular contigs (214) consisted of numerous very short repeats (Supplementary Figures S1B,C), possibly representing random hexamer-based template independent products (Holbrook et al., 2005; Hutchison et al., 2005). Regardless of the origin, the contigs with extensive repeats and inverted repeats do not provide valuable information on gene-carrying plasmids in the environment. Since the sample is known to contain many hundreds of circular MGEs and only has approximately 1% chromosomal DNA, the low number of sequences that could be identified as circular points to severe problems in using MDA mobilome samples as input of PacBio sequencing.

The power of single molecule long read sequencing is indisputable and nicely illustrated by a recent paper identifying 21 large plasmids up to 320 kb in length from cultured, hospital-derived bacterial isolates (Conlan et al., 2014), using paired end information to close plasmids. Here, an impressive sequencing effort proves that large plasmids, scarred by recombination events and with lots of repeated structures, can be completely assembled with technology available today. Critical here is the cultured nature of samples and the massive sequencing effort of four PacBio RSII runs per bacterial isolate in addition to Miseq and 454 sequencing and OpGen optical mapping (OpGen, Inc., Gaithersburg, MD, USA) to confirm results. Considering the substantial per base cost of PacBio sequencing and the difficulties in obtaining large quantities of pure plasmid DNA from environmental samples, the strategy is only feasible for cultivated organisms at the moment.

## METAGENOMICS SPECIES APPROACH TO PLASMID ASSEMBLY

Very recently, it has been suggested that a co-abundance gene groups (CAGs) approach, where groups of genes are binned based on their abundance across multiple samples, could be used to identify plasmids (Nielsen et al., 2014). While this promising approach does not resolve the issue of repeated structures beyond scaffolding, it could allow for high quality scaffolds of plasmids to be analyzed. This could be a useful addition to the plasmid reference datasets liberating them from the current culturing bias, but at the same time introducing the uncertainty of not having intact, experimentally characterized sequences. Based on co-dependency of groups, valuable information on host relations of plasmids could be determined, although it is presumably most efficient for narrow host range plasmids. The weaknesses of the plasmid metagenomic species approach are the dependence on multiple samples and the enormous background of chromosomal DNA, necessitating very deep sequencing in complex samples. Further, while CAGs provide probability of contig coherence, it is not physical and irrefutable proof.

## SUMMARY AND OUTLOOK

For reasons mentioned above, two main routes are realistic for mobilome studies in the coming years; if long read technology approaches the per-base cost of Illumina sequencing, it is possible that untreated metagenomes can be assembled into complete genomes and plasmids. Alternatively, researchers must fine-tune the sample preparation methods to avoid introducing the size bias of MDA to allow complete large plasmids to be analyzed, possibly by removing small plasmids from the pool prior to MDA. Either way, future bioinformatical pipelines must be designed for and constantly take into account the dynamic and circular nature of many bacterial extrachromosomal MGEs. A major factor in this would be for assembly programs to trivially resolve repeats where read pairs are bridging a number of contigs, effectively pushing the boundary of repeat disruption of complete plasmid assembly from the length of a read to the length that a read pair spans.

The study of mobilomes of complex samples has come a long way over the last few years, from PCR and culturing based approaches to analysis using high throughput sequencing and intricate bioinformatics pipelines to expose a world of plasmids and circular elements that previously went undetected. While far from exhaustive, these studies are paving the way for a more complete understanding of the diversity of plasmids and small circular elements, while also highlighting obstacles that are to be overcome with elegant experimental and bioinformatical setups before the complete plasmid pools of environments can be explored and their significance for the survival and evolution of hosts and communities can be mapped.

## Conflict of Interest Statement

The authors declare that the research was conducted in the absence of any commercial or financial relationships that could be construed as a potential conflict of interest.
