# Supplementary material for: Current strategies for mobilome research
Source: Front Microbiol. 2015 Jan 22;5:750. doi: 10.3389/fmicb.2014.00750 (PMC4302988; doi:10.3389/fmicb.2014.00750)
Supplement: Supplementary file 1 [file Data_Sheet_1.DOCX]

***Supplementary Material***

**Current strategies for mobilome research**

**Tue Sparholt Jørgensen^1^, Martin Asser Hansen^1^, Anne Sofie Kiil^1^, Søren Johannes Sørensen^1*^, Lars Hestbjerg Hansen^2*^**

^1^Section of Microbiology, Institute of Biology, faculty of Science, University of Copenhagen, Copenhagen, Denmark

^2^Environmental microbiology & biotechnology, Department of Environmental Science, Aarhus University, Roskilde, Denmark

*** Correspondence:** Lars Hestbjerg Hansen, Environmental microbiology & biotechnology, Department of Environmental Science, Aarhus University, Frederiksborgvej 399, Roskilde, 4000 Roskilde, Denmark. lhha@envs.au.dk

Søren Johannes Sørensen, Section of Microbiology, Institute of Biology, Faculty of Science, University of Copenhagen, Universitetsparken 15, Building 1, 1^st^ floor, Copenhagen, 2100Kbh Ø, Denmark. sjs@bio.ku.dk

1. **Supplementary materials and methods**

Sample preparation, PacBio sequencing and initial bioinformatical processing.

The previously described MDA *Rattus norvegicus* cecum mobilome sample from (Jørgensen et al., 2014) was submitted to PacBio RS sequencing by GATC Biotech, Konstanz, Germany. All subsequent bioinformatics analysis was done in a UNIX environment running the Biopieces framework (Martin Asser Hansen, unpublished, www.biopieces.org). The resulting reads were error corrected in the PacBiotoCA (error correction via Celera Assembler)(Koren et al., 2012) pipeline using the Illumina HiSeq2000 2x100nt reads also used in (Jørgensen et al., 2014) with standard parameters, except for a Maxgap switch of 500 allowing sections of long pacbio reads not covered by illumina data to not be split into individual contigs. The reads were then assembled to contigs with Mira 4.0, allowing 10% megahubs (Chevreux et al., 1999).

## Analysis of circular contigs and data visualization.

The circularization pipeline published in (Jørgensen et al., 2014) was used to identify circular contigs that were then manually assessed by dotplot inspection. Dotplots were created by plotting each sequence against itself using mummer (Kurtz et al., 2004) with the following parameters: -c -L -F -l 12 -maxmatch -n –b, only plotting ATGC characters thus allowing unidentified bases (N’s) to be blank in the plot (Figure 1 B, middle plot).

**Supplementary figure 1**

A

B

C


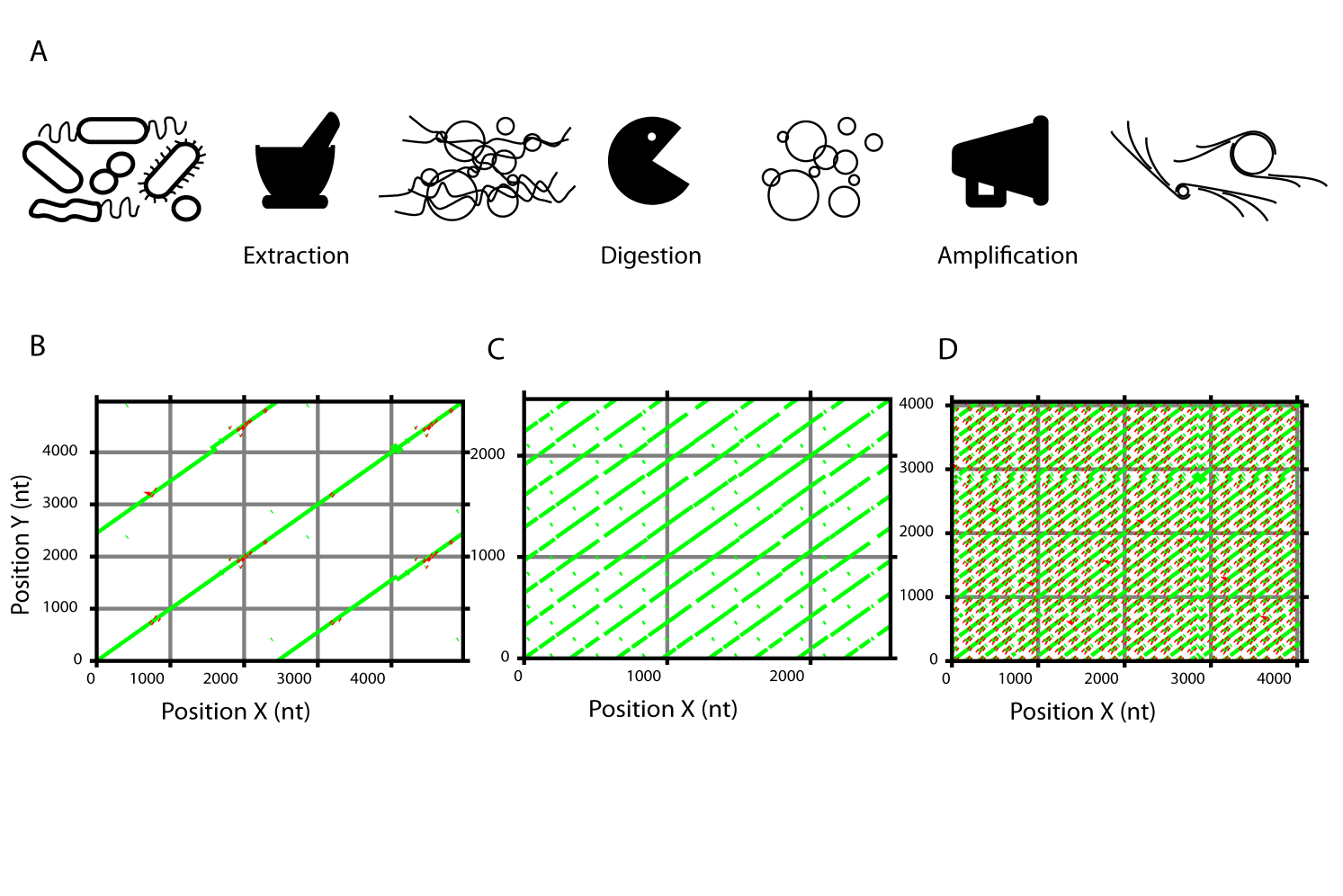
Figure 1. Dot-plots of three illustrative contigs from assembly of PacBio reads matched against themselves. The green colored sections specifies identical positions nucleotide (nt) x and nt y on the sequence whereas the red colored sections specifies positions of nt x and nt y with reverse identical matches. Thus, a circular sequence linearized and multiplied by MDA would have diagonal and parallel green lines with a horizontal distance between them indicating the length of the circular sequence as is seen in A, where a sequence of approx. 5kb with a single repeat of approx. 2.5kb is seen, as would be expected from a circular sequence of 2.5kb. B and C: examples of contigs consisting of multiple short repeats, often with sections with reverse identical matches (red). These types of structures are plausible results of template independent product arising from random hexamer based rolling circle amplification.

1. **References**

Chevreux, Bastien, Wetter, Thomas, and Suhai, S. Genome sequence assembly using trace signals and additional sequence information. German Conference on Bioinformatics , 45-56. 1999.

Ref Type: Conference Proceeding

Jørgensen,T.S., Xu,Z., Hansen,M.A., Sørensen,S.J., and Hansen,L.H. (2014). Hundreds of Circular Novel Plasmids and DNA Elements Identified in a Rat Cecum Metamobilome. PloS one *9*, e87924.

Koren,S., Schatz,M.C., Walenz,B.P., Martin,J., Howard,J.T., Ganapathy,G., Wang,Z., Rasko,D.A., McCombie,W.R., and Jarvis,E.D. (2012). Hybrid error correction and de novo assembly of single-molecule sequencing reads. Nature biotechnology *30*, 693-700.

Kurtz,S., Phillippy,A., Delcher,A.L., Smoot,M., Shumway,M., Antonescu,C., and Salzberg,S.L. (2004). Versatile and open software for comparing large genomes. Genome Biology *5*, R12.
